# Electrode Potential Dependency of Single-Cell Activity Identifies the Energetics of Slow Microbial Electron Uptake Process

**DOI:** 10.3389/fmicb.2018.02744

**Published:** 2018-11-13

**Authors:** Xiao Deng, Akihiro Okamoto

**Affiliations:** ^1^Department of Applied Chemistry, School of Engineering, The University of Tokyo, Tokyo, Japan; ^2^International Center for Materials Nanoarchitectonics, National Institute for Materials Science, Tsukuba, Japan; ^3^Center for Functional Sensor and Actuator, National Institute for Materials Science, Tsukuba, Japan

**Keywords:** extracellular electron transfer, sulfate-reducing bacteria, whole-cell electrochemistry, nanoscale secondary ion mass spectrometry, anabolism, energy metabolism, transcriptome, oligotrophic environments

## Abstract

Electrochemical measurements have been widely applied to study microbial extracellular electron transport processes. However, because electrochemistry detects not only microbial electron transport but also other reactions, background signals comparable to or larger than microbial ones hamper the identification of microbial electrochemical properties. This problem is crucial especially for the detection of electron uptake processes by slow-growing microbes in low-energy subsurface sediments, as the environmental samples contain electrochemically active humus and mineral particles. In this study, we report a cell-specific stable isotope analysis to quantify the electrode potential dependency of anabolic activity in individual cells for identifying the electron uptake energetics of slow-growing bacteria. Followed by the incubation of *Desulfovibrio ferrophilus* IS5 cells with isotopic ^15^N-ammonium as the sole *N* source on electrodes poised at potentials of -0.2, -0.3, -0.4, and -0.5 V [vs. standard hydrogen electrode (SHE)], we conducted nanoscale secondary ion mass spectroscopy (NanoSIMS) to quantify ^15^N assimilation in more than 100 individual cells on the electrodes. We observed significant ^15^N assimilation at potentials of -0.4 and more ^15^N assimilation at -0.5 V, which is consistent with the onset potential for electron uptake via outer-membrane cytochromes (OMCs). The activation of cell energy metabolism was further examined by transcriptome analysis. Our results showed a novel methodology to study microbial electron uptake energetics. The results also serve as the first direct evidence that energy acquisition is coupled to the electron uptake process in sulfate-reducing bacteria that are ubiquitous in the subsurface environments, with implications on the electron-fueled subsurface biosphere hypothesis and other microbial processes, such as anaerobic iron corrosion and anaerobic methane oxidation.

## Introduction

Vast and active microbial biospheres have been discovered in subsurface environments with scarce energy sources (Parkes et al., 1994; Fredrickson and Onstott, 1996; D’Hondt et al., 2004, 2015). Although these environments lack sufficient soluble electron donors, they preserve abundant minerals, such as iron sulfides (FeS), and different redox states to generate a flow of natural electrical currents. Through mineral oxidation or bridging different redox states, these environments thermodynamically provide sufficient energy to generate reduced nicotinamide adenine dinucleotide (phosphate) [NAD(P)H] to maintain cell metabolic activity (Sandstrom et al., 2002; Nakamura et al., 2010; Nielsen et al., 2010). Therefore, direct electron extraction from extracellular solids, namely extracellular electron uptake, has been proposed to be crucial for the understanding of the microbial energy acquisition in low-energy (oligotrophic) subsurface environments (Deng et al., 2018). Extracellular electron uptake has been recognized in several strains, including iron-reducing and -oxidizing bacteria (Gregory et al., 2004; Strycharz et al., 2008; Rabaey and Rozendal, 2010; Okamoto et al., 2014; Beese-Vasbender et al., 2015; Ishii et al., 2015; Choi and Sang, 2016; Rowe et al., 2018). Furthermore, recent studies have identified widespread electron uptake conduits, outer-membrane multi-heme cytochromes (OMCs), in sedimentary microbes, and thus indicated the ubiquity of the electron uptake process in subsurface environments (Deng et al., 2018). Therefore, microbial utilization of natural electrical currents may explain how microbes survive in oligotrophic subsurface environments.

Amperometry (current-time measurements) and voltammetry (current measurements during potential scanning) are the most widely used methods to elucidate the microbial extracellular electron transport process and energetics. However, because the measured currents are a sum of background and microbial signals, it is difficult to identify biogenic signals when background currents are comparable to or larger than the microbial currents (Venzlaff et al., 2013; Rowe et al., 2014; Deng et al., 2015). Furthermore, wild-type cells and OMCs-deficient mutants (Richter et al., 2009; Carmona-Martinez et al., 2011), or the wild-type cells adjusted to express different amounts of electron-uptake components (Deng et al., 2018) are required to assign redox signals to microbial electron-uptake components (e.g., OMCs) in the voltammetry. Thus, the assignment of voltammetric signals to the microbial electron transport reaction has been challenging, especially in regards to slow-growing microbes in sediment samples, which contain complex redox active compositions, such as the humic particles, and most likely generate background currents larger than microbial signals on both amperometry and voltammetry. Moreover, as most sediment microbes remain uncharacterized and, thus, genetic mutant strains and the conditions to control the expression for the electron-uptake conduits have yet to be developed, the assignment of signals to microbial electron uptake would also be difficult (Nealson, 1997; Jorgensen and Marshall, 2016; Krukenberg et al., 2016). In this study, we aim to develop a methodology to specifically monitor microbial processes coupled with electron uptake.

Nanoscale secondary ion mass spectrometry (NanoSIMS) detects cellular isotopic ratios, such as ^13^C/C_total_ and ^15^N/N_total_, at high sensitivity (<0.05% isotopic ratio resolution) and high spatial resolution (<50 nm) (Musat et al., 2012; Yang et al., 2015). NanoSIMS, coupled with isotopic cell labeling through incubations with ^15^N-nitrogen and ^13^C-carbon sources, has been applied to analyze slow microbial metabolic processes at single-cell level, such as anaerobic methane oxidation (Orphan et al., 2009; McGlynn et al., 2015; Marlow et al., 2016) and nitrogen assimilation by deep-subsurface microbes (Morono et al., 2011; Inagaki et al., 2015). Because microbial electron uptake is also expected to associate with energy production and activate cellular metabolism, ^15^N assimilation could serve as a specific indicator of the electron uptake process. Here, we analyzed the dependency of cell activity on the electrode potential to identify the electron uptake process and its energetics independently from background currents. *Desulfovibrio ferrophilus* IS5 served as the model microbe because its electron uptake capability and energetics have been elucidated in a previous study (Deng et al., 2018). We used amperometry to incubate cells on electrodes poised at different potentials without H_2_ generation and ^15^N ammonium chloride serving as the sole *N* source. Subsequently, we analyzed the ^15^N ratio of individual cells incubated on the electrodes to elucidate the onset potential of microbial metabolic activity. The active energy metabolism of cells on the electrodes was further confirmed by transcriptome analysis.

## Materials and Methods

### Cell Cultivation

*D. ferrophilus* IS5 [Deutsche Sammlung von Mikroorganismen (DSM) no. 15579] was precultivated in butyl rubber-stoppered glass vials containing 100 mL of Deutsche Sammlung von Mikroorganismen und Zellkulturen (DSMZ) 195c medium at 28°C with an anoxic headspace of CO_2_/N_2_ (20:80, v/v). After 5 days, a culture containing a small amount of black iron sulfide precipitant was obtained, which was used for electrical incubation.

### Electrical Incubation of Cells on the Electrodes

Electrical incubation was conducted in three-electrode reactors kept in a COY anaerobic chamber filled with 100% N_2_ as previously described (Deng et al., 2018). An anoxic salt medium supplied with ^15^N-labeled ammonium was used as the electrolyte, which had the following composition: 457 mM NaCl, 47 mM MgCl_2_, 7.0 mM KCl, 5.0 mM NaHCO_3_, 1.0 mM CaCl_2_, 1.0 mM K_2_HPO_4_, 25 mM Na^+^-Hepes (pH 7.5), 1 mL of selenite-tungstate solution (12.5 mM NaOH, 11.4 mM Na_2_SeO_3_⋅5H_2_O, and 12.1 mM Na_2_WO_4_ 2H_2_O), 1 mL of trace element solution SL-10 (described in DSMZ medium 320), 21 mM Na_2_SO_4_ as the electron acceptor, 1 mM ^15^N-NH_4_Cl, and 1 mM acetate as the carbon source. Indium tin-doped oxide (ITO) electrodes were poised at potentials between -0.2 and -0.5 V as the sole energy source during the electrical incubation for 2 days. No color and resistance change of ITO electrode were confirmed after the potential poising. *D. ferrophilus* IS5 cells were accumulated from a bottle of 5-day liquid cell culture, resuspended in the ^15^N-labeled anoxic salt medium, and added into the electrochemical reactors to a final OD_600nm_ of 0.3 at 30 min after the current measurement started. As a negative control, cells were added at the same density to glass vials, which lacked electrodes and contained the same salt medium as the electrolyte in the electrochemical reactor. This negative control was referred to as the “open circuit (o.c.) condition.”

### Scanning Electron Microscopy (SEM)

After conducting the electrochemical measurements, the ITO electrodes were removed from reactors, fixed with 2.5% glutaraldehyde, and washed by immersing electrodes three times in 50 mM Na^+^-Hepes (pH 7.4). The washed samples were dehydrated in 25, 50, 75, 90, and 100% ethanol gradients in the same buffer, exchanged three times with *t*-butanol, and freeze-dried under a vacuum. The dried samples were coated with evaporated platinum and viewed using a Keyence VE-9800 microscope.

### Nanoscale Secondary Ion Mass Spectrometry (NanoSIMS)

The ITO electrode samples were prepared based on methodology as previously described (Saito et al., 2017). ITO electrodes with cell attachments were cut into 7 mm × 7 mm pieces to fit the NanoSIMS sample holder. Cells cultivated in glass vials without potential poising (the o.c. condition) were transferred to the surface of 7 mm × 7 mm ITO electrode pieces pretreated with 0.01% (w/v) poly-L-lysine solution. Subsequently, they were subjected to fixation and dehydration as described in the SEM experimental section. The dried cells attached to the ITO electrodes were analyzed using the CAMECA NanoSIMS 50L system. A Cs^+^ beam was irradiated on the sample surface, and the amounts of secondary ions (^12^C^14^N and ^12^C^15^N) were quantified. For the analysis of the isotopic N ratio in individual cells, data were processed by Fiji (Schindelin et al., 2012), and regions of interests (ROIs) were drawn for individual cells to obtain the sum of ^12^C^14^N and ^12^C^15^N signals in each ROIs using the plugin OpenMIMS.

### Transcriptome Analysis

*D. ferrophilus* IS5 cells were cultivated on the surface of -0.4 V-poised ITO electrode or under the o.c. condition for 1 week. After incubation on the electrode, the supernatant in the reactor was discarded using a pipette, and cells attached on the electrodes were homogenized by thoroughly pipetting with Tris-EDTA (TE) buffer (pH 8) containing 2 mg/mL lysozyme on the electrode surface. Subsequently, the cells were transferred to a sterile microtube and proceeded to the RNA extraction procedure using the TAKARA Nucleobond RNA kit following the manufacturer’s instructions. Cells incubated in the glass vial were transferred and homogenized inside a sterile microtube and proceeded to RNA extraction using the same kit. rRNA was removed using a Ribo-Zero magnetic kit (Gram-negative bacteria), and a complementary DNA (cDNA) library was prepared for sequencing using the TruSeq Stranded mRNA Library Prep Kit (Illumina) following the manufacturer’s guidelines. The cDNA library was sequenced on a HiSeq 2500 instrument (Illumina). To compare relative expression patterns of IS5 cells under the electron uptake and o.c. conditions, read counts were quantified using the Fragments Per Kilobase of transcript per Million fragments sequenced (FPKM) metric. The FPKM values were normalized using the housekeeping gene, adenylate kinase (*adk*).

## Results and Discussion

### Electrical Incubation of IS5 Cells Before NanoSIMS Measurement

**FIGURE 1:**
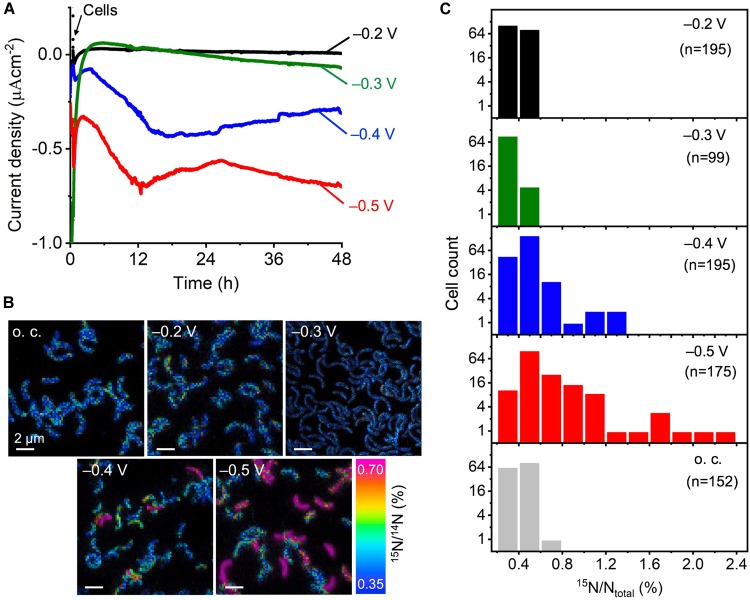
Electrical incubation of *D. ferrophilus* IS5 on ITO electrodes in the presence of ^15^N-NH_4_Cl serving as the sole nitrogen source for NanoSIMS analysis. **(A)** Cathodic current, represented as negative current, vs. time on electrodes poised at different potentials between –0.2 and –0.5 V (vs. SHE). Cells were added to the reactor at 30 min as indicated by black arrow. **(B)** NanoSIMS images, in which the color gradient indicates ^15^N abundance expressed as ^15^N/^14^N (%). **(C)** Histograms of the ^15^N ratio in individual cells inoculated on ITO electrodes poised at different potentials for 48 h.

Anoxic electrochemical reactors equipped with ITO electrodes poised at four different potentials, -0.2, -0.3, -0.4, and -0.5 V, were used for the electrical incubation of *D. ferrophilus* IS5 cells. Followed by the background current stabilization to less than -0.05 μA cm^-2^, we introduced IS5 cells to the electrode surface poised at -0.2 V, and the increase in cathodic current, which is the negative current, was negligible during the following electrical incubation of 48 h (Figure 1 and Supplementary Figure S1). In the case of the -0.3 V-poised electrode, the initial background current was larger than 1 μA cm^-2^ but sharply declined with time, which tendency did not change upon the introduction of IS5 cells onto the electrode, and no significant current production was detected during the following electrical incubation. In contrast, on the -0.4 V-poised electrode and while the background current was less than -0.1 μA cm^-2^, the addition of IS5 cells caused an immediate cathodic current increase and decrease in the first hours. Following, there was a gradual current rise lasting for nearly 12 h, reaching a maximum of >-0.4 μA cm^-2^, and maintaining at >-0.25 μA cm^-2^ during the following incubation. Meanwhile, the -0.5 V-poised electrode had a higher background current around -0.4 μA cm^-2^ compared to the -0.4 V-poised electrode, which was possibly due to the reduction of some medium components. The current profile of the -0.5 V-poised electrode after IS5 cells addition showed a similar increase tendency as the -0.4 V-poised electrode, which reached a maximum of >-0.7 μA cm^-2^ within 12 h and maintained at around >-0.6 μA cm^-2^ during the left incubation period. Because little cathodic current for H_2_ evolution was detected on the ITO electrodes at potentials more positive than -0.9 V (Deng et al., 2015), the observed current production by IS5 cells on the -0.4 and -0.5 V-poised electrodes was assignable to electrons extracted by cells from the electrodes.

### Electrode Potential Dependency of Single-Cell Activity Analyzed by NanoSIMS

To analyze the metabolic Electrical incubationactivity of cells that extracted electrons from the electrode, we used cells that remained attached to the electrode surface, even after washing the electrode surface. As observed by SEM and NanoSIMS, direct cell attachments on the electrodes were observed at all potentials (Supplementary Figure S2 and Figure 1B). We visualized the ^15^N/^14^N ratio in the individual cells attached to the electrode surface and found that cells on the -0.2 and -0.3 V-poised electrodes had comparable ^15^N/^14^N ratios to cells under the negative o.c. condition, which was also comparable to the natural abundance of ^15^N. In contrast, some cells on the -0.4 V showed clearly higher ^15^N/^14^N ratios around 0.7%, and the number of cells with high ^15^N/^14^N ratios further increased on the -0.5 V-poised electrode. Because cells produced currents on the -0.4 and -0.5 V-poised electrodes, these results suggest that cell metabolism was activated by electron uptake from the electrodes.

We analyzed the cell number distribution of isotopic ^15^N ratio (^15^N/N_total_) on the electrodes poised at different potentials in the histogram (Figure 1C). The isotopic ^15^N ratio of IS5 cells on the -0.2 and -0.3 V-poised electrodes was narrowly distributed within 0.6%, with an average ^15^N ratio of 0.41 and 0.37%, respectively, which did not differ much from the ^15^N ratio distribution of cells under the o.c. condition (average ^15^N/N_total_ of 0.41%) or the natural abundance of ^15^N (0.37%). In contrast, cells on the -0.4 V-poised electrode had a wider distribution of ^15^N ratio extending to over 1% and a higher average ^15^N ratio of 0.46%. Meanwhile, cells on the -0.5 V-poised electrode showed a wider distribution of ^15^N ratio than cells on the -0.4 V-poised electrode, which extended to >2% and had the highest average ^15^N ratio of 0.63%.

**FIGURE 2:**
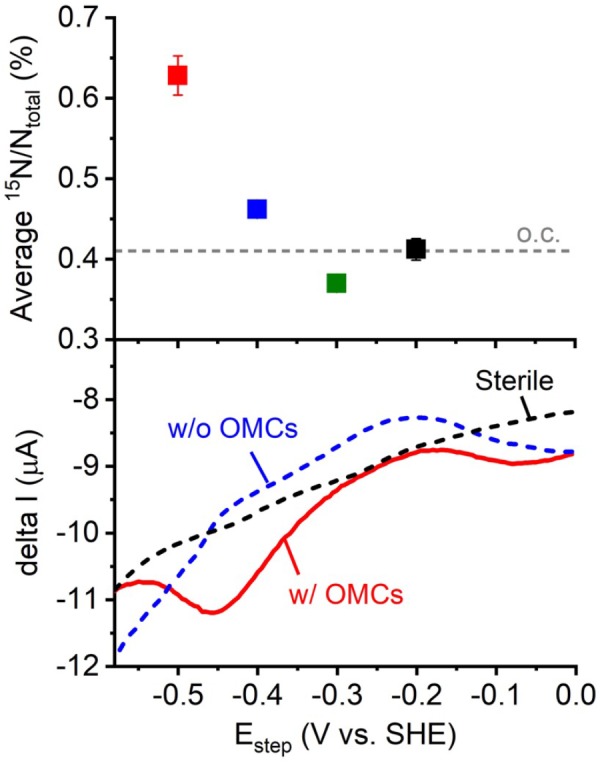
Plot of the average ^15^N/N_total_ measured by NanoSIMS against electrode potential (Figure 1) and the electron uptake current measured by differential pulse voltammetry (DPV) with potential scanned toward the negative direction and pulse increment, 5 mV; pulse amplitude, 50 mV; pulse width, 300 ms; and pulse period, 5 s (Deng et al., 2018). OMCs represents outer-membrane cytochromes, which are the cell surface redox proteins that mediate electron uptake process of *D. ferrophilus* IS5 cells. Differential pulse voltammograms of the IS5 cells with high (w/ OMCs) and low expression (w/o OMCs) of OMCs, and sterile medium were shown.

We plotted the average cellular ^15^N ratio to the electrode potentials from -0.2 to -0.5 V to compare the electrode potential dependency of cellular activity with the previously reported IS5 electron uptake energetics. As shown in Figure 2, the average ^15^N ratio of cells on electrodes poised at -0.4 and -0.5 V was higher than electrodes poised at potentials more positive than -0.3 V, indicating that the onset potential of electron uptake by IS5 was between -0.3 and -0.4 V. This onset potential determined by NanoSIMS was consistent with that determined by a previous differential pulse voltammetry (DPV) study of IS5 cells that overexpressed OMCs to extract electrons from the electrodes (Deng et al., 2018). Furthermore, while the DPV also detected a broad reduction signal at potentials more positive than -0.3 V, which was previously assigned to the reduction of FeS species, the onset potential determined by NanoSIMS clearly eliminated the possibility of efficient electron uptake associated with the reduction of FeS in *D. ferrophilus* IS5. These results demonstrate that analysis of the dependency of cell activity on electrode potentials can identify the energetics of the primary electron uptake process independently from the background currents.

### Transcriptome Analysis of Cells Under Electron Uptake and the o.c. Condition

**FIGURE 3:**
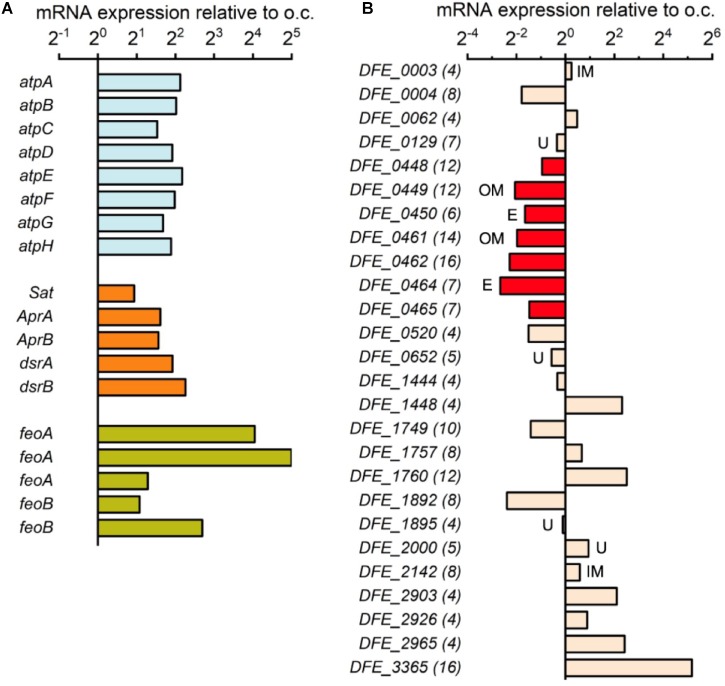
Gene expression of incubated *D. ferrophilus* IS5 on the –0.4 V-poised electrode relative to the open-circuit (o.c.) condition. **(A)** Expression of genes encoding F-type ATPase (*atpA-H*), dissimilatory sulfate reduction (*Sat, AprA/B*, and *dsrA/B*), and ferrous ion importer (*feoA/B*). **(B)** Expression of 26 genes encoding cytochromes with 4 or more heme-binding motifs in the IS5 genome. The number of heme-binding motifs is shown in parentheses, and the predicted localization of proteins is shown next to the bar (IM, inner membrane; OM, detected in outer membrane; U, unknown; blank, periplasm).

To further confirm that *D. ferrophilus* IS5 cells have active energy metabolism during the electron uptake process, we conducted transcriptome analysis of IS5 cells incubated on the -0.4 V-poised electrode and under the o.c. condition to compare the expression of genes encoding energy metabolism during electron uptake. To enhance and suppress gene expression for cells adapted to the electron uptake condition and preculture condition, respectively, we used cells with an extended 1-week incubation period. Incubation on the -0.4 V-poised electrode enabled us to confirm the activation of cell metabolism by a minimum amount of energy during the electron uptake, as -0.4 V was the most positive potential with detectable microbial current production and active metabolism (Figure 1). When cells conducted electron uptake from the electrode, 2127 genes out of the total 3431 genes (ca. 62%) were overexpressed in IS5 compared to the o.c. condition. Additionally, while approximately 17% of total genes were less than 10 FPKM under the o.c. condition, only 2% of total genes were scarcely expressed in the presence of the -0.4 V-poised electrode. Notably, genes encoding central energy metabolism, including F-type ATPase and dissimilatory sulfate reduction as annotated by KEGG Mapper (Kanehisa et al., 2016), were overexpressed approximately fourfold compared to cells under the o.c. condition (Figure 3A), which was comparable to the gene expression by SRB cells in active-growing exponentionary phase relative to non-growth stationary phase (Zhang et al., 2006). These results agreed with the notion that IS5 cells obtained energy during electron uptake from electrodes.

The expression of genes encoding cytochromes was also investigated to understand the roles of cytochromes in the electron uptake process. Previously, 26 genes encoding multi-heme cytochromes have been identified in the IS5 genome (Deng et al., 2018), which included two OMCs clusters (from *DFE_448* to *DFE_450* and from *DFE_461* to *DFE_465*) mediating extracellular electron uptake in IS5. However, both OMCs clusters were downregulated nearly fourfold under the electron uptake condition compared to the o.c. condition (Figure 3B). The overexpression of genes encoding energy metabolism but downregulation of genes encoding OMCs suggests that cells might require more metabolic machinery rather than an increased number of OMCs at the time point when RNA was extracted from cells after the 1-week incubation.

Although no expression pattern exists for genes encoding other cytochromes localized in the periplasm or on the inner membrane (Figure 3B), a 16-heme periplasmic cytochrome encoded by the gene *DFE_3365* was significantly overexpressed nearly 36-fold under the electron uptake condition. This result suggests that a periplasmic cytochrome network may be formed during electron uptake to store and pass electrons received from OMCs to other cellular redox proteins and mediators or that this periplasmic cytochrome is unstable during the electron uptake process. Alternatively, this periplasmic cytochrome might attach to the outer membrane and engage in extracellular electron uptake instead of those shown to be important in a short period experiment in a previous report (Deng et al., 2018). Notably, all five genes encoding ferrous ion importer in the IS5 genome were significantly overexpressed up to 32-fold under the electron uptake condition (Figure 3A), suggesting that cells had a high requisite for ferrous ion, which may be used for the synthesis of proteins with iron centers, such as cytochromes.

While we observed a similar amounts of electron extraction from ITO electrodes poised at -0.4 and -0.5 V, cells at -0.5 V assimilated significantly more ^15^N ammonium than those at -0.4 V (Figure 1 and Supplementary Figure S1), which is not likely to occur if cells shared the same mechanism for energy conservation under these two conditions. Because the electrode potential induced an alteration of active metabolic pathways in *Shewanella* species (Lian et al., 2016; Hirose et al., 2018), -0.5 V may activate pathways which are more efficient in energy conservation coupled with electrode uptake in IS5. Although the -0.5 V is likely harder to be achieved than -0.4 V in natural environments, this metabolism shift would be an important mechanism to understand the ecological and physiological roles of the microbial electron uptake process in energy-limited sediments.

In this study, we developed a novel methodology to identify the energetics of microbial electron uptake processes, which combined electrical incubation of cells on electrodes at different potentials by amperometry and potential dependency analysis of the cell activity on electrodes by NanoSIMS. Our results showed that the electrode potential dependency of cell activity data was able to identify the electron uptake energetics in *D. ferrophilus* IS5, while its voltammetry data had significant background currents. The combination of our method with other single-cell analysis techniques, such as fluorescent *in situ* hybridization (FISH), would highly advance our understanding of slow microbial extracellular electron uptake processes and energetics in the complex environmental sediment samples. In addition, this study provides the first piece of evidence that the metabolic activity of SRB is sustainable by a minimum amount of energy obtained via electron uptake from electrodes, which supports the novel hypothesis of subsurface microbes fueled by direct electron uptake from reduced minerals.

## Data Availability Statement

All data needed to evaluate the conclusions in the paper are present in the paper and/or the Supplementary Materials. Additional data related to this paper may be requested from the authors.

## Author Contributions

XD and AO devised the study. XD conducted the experiments and analyses. All authors contributed to the data interpretation and writing of the manuscript.

## Conflict of Interest Statement

The authors declare that the research was conducted in the absence of any commercial or financial relationships that could be construed as a potential conflict of interest.
