# Supplementary material for: Electrode Potential Dependency of Single-Cell Activity Identifies the Energetics of Slow Microbial Electron Uptake Process
Source: Front Microbiol. 2018 Nov 13;9:2744. doi: 10.3389/fmicb.2018.02744 (PMC6243204; doi:10.3389/fmicb.2018.02744)
Supplement: Supplementary file 1 [file Data_Sheet_1.docx]

Supplementary Material

**Electrode Potential Dependency of Single-Cell Activity Identifies the Energetics of Slow Microbial Electron Uptake Process**

**Xiao Deng^1,2^, Akihiro Okamoto^2,3*^**

*** Correspondence:** OKAMOTO.Akihiro@nims.go.jp

## Supplementary Figures


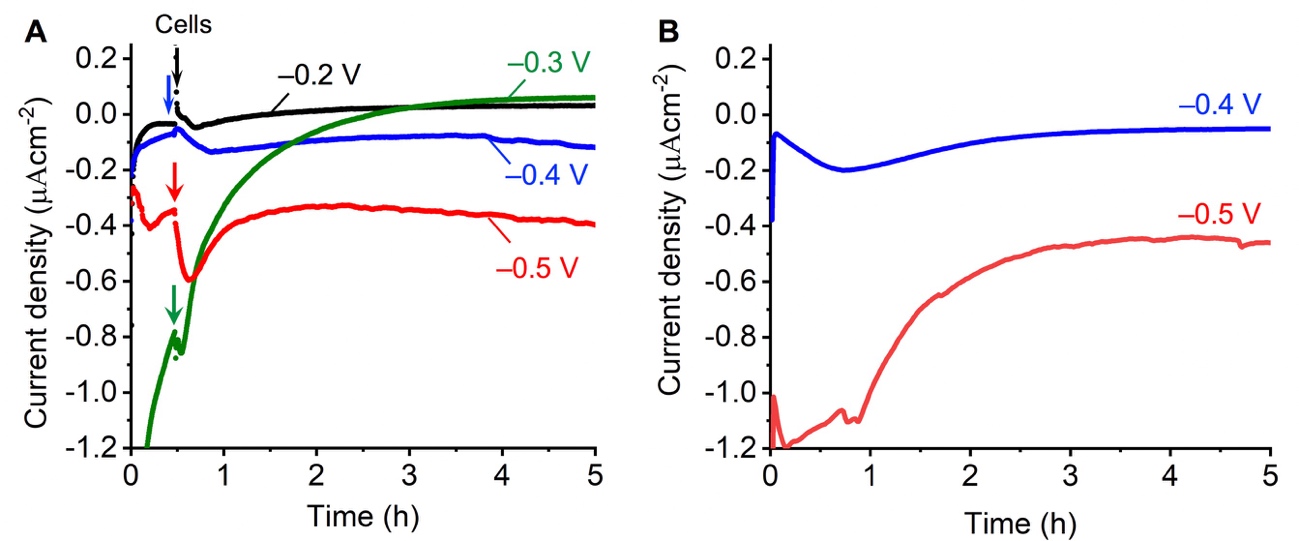


**Supplementary Figure 1** (A) Current production vs. time enlarged from Figure 1 panel A. *D. ferrophilus* IS5 cells were introduced at the timing of arrows to electrodes poised at different potentials between –0.2 and –0.5 V (vs. SHE) at 30 minutes. (B) Background currents generated on the ITO electrodes poised at –0.4 V and –0.5 V with sterile medium, which stabilized around –0.06 µA cm^-2^ and –0.43 µA cm^-2^, respectively.


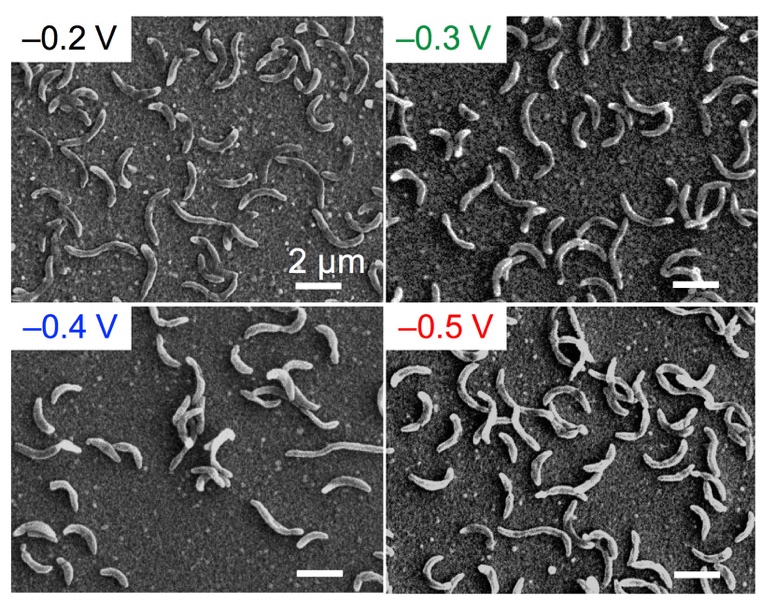


**Supplementary Figure 2** Scanning electron microscopy images of *D. ferrophilus* IS5 cells attached to the surface of ITO electrodes after incubation at potentials of –0.2, –0.3, –0.4 and ­–0.5 V (vs. SHE) for 1 week.
